# Size-Effect-Based Forming Behavior and Multi-Objective Die Optimization of Metallic Fuel Cell Bipolar Plates

**DOI:** 10.3390/ma19163519

**Published:** 2026-08-19

**Authors:** Jianbin Zhu, Shusheng Liu, Chao Ma, Siming Wang, Yuanding Cheng, Tao Wang, Jianghan Zhong, Yang Yang, Feng Xu

**Affiliations:** 1 Shandong Tietou Energy Investment Group Co., Ltd., Jinan 250000, China; a394752058@126.com (J.Z.); 18678827283@126.com (S.L.); a18668988356@126.com (C.M.); a506760207@126.com (T.W.); 2 Shandong Tiefa New Energy Co., Ltd., Jinan 250000, China; 18354891877@126.com (S.W.); a80817004@126.com (Y.C.); 3 College of New Energy, China University of Petroleum (East China), Qingdao 266580, China; 4 College of Mechanical and Electrical Engineering, Shandong University of Science and Technology, Qingdao 266590, China; 5 Beijing Wei Yi Technology Research Institute Co., Ltd., Beijing 100144, China

**Keywords:** size effect, fuel cell bipolar plates, thinning rate, springback angle, multi-objective optimization

## Abstract

Ultra-thin metal bipolar plates are critical components of proton exchange membrane fuel cells (PEMFCs), and their forming characteristics decisively influence service performance. This study proposes a constitutive model incorporating size effects to elucidate how sheet thickness and grain size govern stress–strain responses and formability of ultra-thin plates. The verified model is employed in finite element analysis for formability of ultra-thin plates. Based on the results of simulation, key stamping die parameters were optimized using Random Forest and XGBoost surrogate models. Results indicate that increasing grain sizes reduces grain boundary density, leading to stress localization within coarse grains and promoting local thinning. This effect increases stored elastic energy and simultaneously raises the maximum stress, thinning rate, and springback angle. Conversely, the increasing sheet thickness strengthens triaxial constraint and raises forming stress, while suppressing thinning and springback through enhanced strain redistribution and plastic dissipation. Thus, grain coarsening degrades formability overall, whereas increasing thickness introduces a trade-off between higher forming stress and improved dimensional stability. Both surrogate models demonstrated high predictive accuracy on unseen data (maximum error is 2.01%), identifying a non-standard parameter combination (*α* = 16.0°, *R* = 0.30 mm, *h* = 0.48 mm, *W* = 1.46 mm, *S* = 0.73 mm) that yields a thinning rate of 4.43% and a springback angle of 0.151°, a level of precision that is difficult to achieve using conventional orthogonal experimental design. This result was verified by additional finite element simulations. The proposed constitutive model and optimization approach provide a theoretical framework and practical guideline for micro-scale bipolar plate die design.

## 1. Introduction

The metallic bipolar plate is a core component of proton exchange membrane fuel cells (PEMFCs), accounting for approximately 60% of the stack volume. It serves to connect individual cells in series, separate fuel from oxidant gases, and provide mechanical support [1]. While microscopic innovations such as heterointerface catalysts [2] and single-atom catalysts [3] have significantly enhanced electrochemical performance, translating these advances into system-level benefits critically depends on the macroscopic components. Specifically, the forming accuracy of ultra-thin metallic bipolar plates significantly influences electrical contact resistance, gas sealing integrity, and long-term durability [4,5]. In plates with thicknesses of 0.05–0.1 mm, only a few grain layers exist through the thickness; consequently, variations in grain size and crystallographic orientation induce significant mechanical anisotropy [6]. The dimensional accuracy of the stamped flow channels directly governs the interfacial contact resistance between the bipolar plate and the gas diffusion layer, the uniformity of reactant gas distribution, and the integrity of the sealing interface. Consequently, minimizing thickness variation and springback during the stamping process is essential for ensuring the electrochemical performance and long-term durability of PEMFC stacks. However, existing theoretical models rarely account for the coupled effects of sheet thickness and grain size on stress–strain responses during forming, and the influence of die geometry parameters on forming accuracy remains insufficiently understood. Therefore, reliably predicting forming-induced stress and deformation, combined with rational optimization of die parameters, is essential for guiding the stamping process design of ultra-thin metallic bipolar plates for PEMFC applications.

Various forming techniques, including hydroforming, rubber pad forming, and conventional stamping, have been extensively investigated for the manufacturing of metallic bipolar plates. Among these methods, stamping has emerged as the predominant industrial choice due to its high production efficiency, low operational cost, and inherent process simplicity [4,7]. Despite these advantages, the stamping of ultra-thin metal sheets is inherently susceptible to multiple forming defects, including excessive localized thinning, fracture at micro-channel corners, wrinkling, and springback, all of which severely compromise the dimensional accuracy and long-term service performance of the final product. To address these challenges, extensive research efforts have been dedicated to understanding the influence of die geometric parameters and process variables on forming quality. Bong et al. [8] and Zhang et al. [9] demonstrated that multi-stage stamping processes can optimize stress and strain distribution while improving the aspect ratio of micro-channels; Li et al. [10] revealed a significant nonlinear coupling effect between die clearance and fillet radius that must be carefully balanced; Khatir et al. [11] and Bong et al. [9] confirmed that increasing the fillet radius effectively reduces the maximum thinning rate and enhances achievable forming depth; and Acar et al. [12] and Seddighi et al. [13] showed that optimized blank holder designs can effectively suppress wrinkling while improving thickness uniformity across the formed part. In terms of forming accuracy prediction, Hu et al. [14] and Modanloo et al. [15] demonstrated that the finite element method (FEM) is a widely employed and efficient tool for evaluating thickness distribution and predicting fracture initiation, and Tran et al. [16] developed hybrid optimization models combining artificial neural networks with genetic algorithms that demonstrate robust capabilities in tuning process parameters. Nevertheless, springback, defined as the elastic recovery resulting from the release of non-uniformly distributed residual stresses after load removal [17], remains a critical bottleneck limiting dimensional precision. This challenge is particularly pronounced in the micro-plastic forming of ultra-thin sheets with a thickness of 0.05 to 0.1 mm, where only a few grain layers exist through the thickness direction. The resulting size effect, strongly influenced by the ratio of sheet thickness to average grain size (t/d), significantly alters the mechanical response of the material [18], and Diehl et al. [19] demonstrated that conventional macroscopic constitutive models are inadequate for accurately predicting micro-scale springback behavior [20]. In summary, while existing studies by Kolahdooz et al. [21], Mohammadtabar et al. [22], Huang et al. [23], and Zhang et al. [24] have collectively established a valuable empirical foundation for bipolar plate forming, a systematic understanding of the coupled effects of die geometry and process parameters on both thinning and springback under size-effect conditions for ultra-thin 304 stainless steel bipolar plates remains notably lacking. There is a growing demand for developing a high-fidelity prediction framework and a rational parameter optimization strategy specifically tailored to micro-stamping processes.

Building on this foundation, researchers have employed various optimization strategies to improve the forming quality of metallic bipolar plates. Response surface methodology (RSM), integrated with finite element analysis (FEA) and damage criteria, has been widely adopted to systematically identify the dominant process parameters and establish predictive models for key performance indicators. These studies consistently reveal that die corner radius, clearance, and friction coefficient are the primary determinants of thinning and filling depth, while stamping speed exerts a relatively minor influence [25,26]. The resulting regression models have demonstrated high predictive accuracy for filling rate and springback, facilitating the fabrication of deep flow channels in ultra-thin stainless steel sheets [27,28]. However, RSM relies on low-order polynomial approximations, which inherently limits its capacity to capture highly nonlinear and strongly coupled relationships among process variables. The Taguchi method has also been applied to evaluate the coupled effects of die geometry and process conditions on forming quality, confirming that corner radius, channel geometry, and blank holder force are the dominant factors governing thinning rate and springback [29,30,31]. While robust for screening important factors, this method is restricted to discrete factor levels and does not support continuous design-space exploration or gradient-based refinement. To overcome these limitations, meta-heuristic algorithms have been introduced. Coupling genetic algorithms (GA) with RSM reduced prediction errors by approximately 53% compared with RSM alone [32], and comparative analyses of multiple meta-heuristic approaches have further confirmed the robustness of GA in handling complex parameter spaces [33]. Nevertheless, GA-based methods often rely on RSM-fitted surrogate models, thereby inheriting the polynomial approximation limitations of the underlying response surface. In a related effort, Yu et al. [34] proposed a multi-objective particle swarm optimization (MOPSO) framework targeting simultaneous minimization of thickening and thinning rates. Despite their predictive capabilities, those methods are constrained by low-order polynomial assumptions whose high nonlinearity and strong coupling are inherently limited. Furthermore, it is mainly confined to single- or simple bi-objective optimizations, lacking efficient strategies for high-dimensional synergistic optimization of metrics such as thinning and springback. Consequently, it is urgent to introduce advanced algorithms that combine robust nonlinear mapping with high computational efficiency, thereby overcoming the bottlenecks in complex multi-objective forming quality control.

Despite the accumulation of experimental and finite element (FE) data, two critical challenges remain in the precision forming of metallic bipolar plates for PEMFCs. First, while intrinsic size effects, arising from the comparable scales of sheet thickness and grain size, are well-documented in fundamental micro-forming [6], they are often simplified or decoupled from practical multi-objective optimization frameworks for complex channel geometries. Second, the non-linear coupling effects between die geometric parameters under these size-dependent constraints have not been systematically characterized. To address these gaps, this study integrates the surface layer model and composite layer model to elucidate size-dependent deformation mechanisms during stamping. Subsequently, Random Forest (RF) and XGBoost surrogate models are employed to capture these complex mappings. The primary objective is to determine the optimal trade-offs among multiple objectives by comprehensively considering key die geometric parameters, including punch fillet radius, die fillet radius, channel depth, and channel width. This result is validated through additional finite element simulations.

## 2. Experimental Details

### 2.1. Materials and Heat Treatment Tests

**Figure 1:**
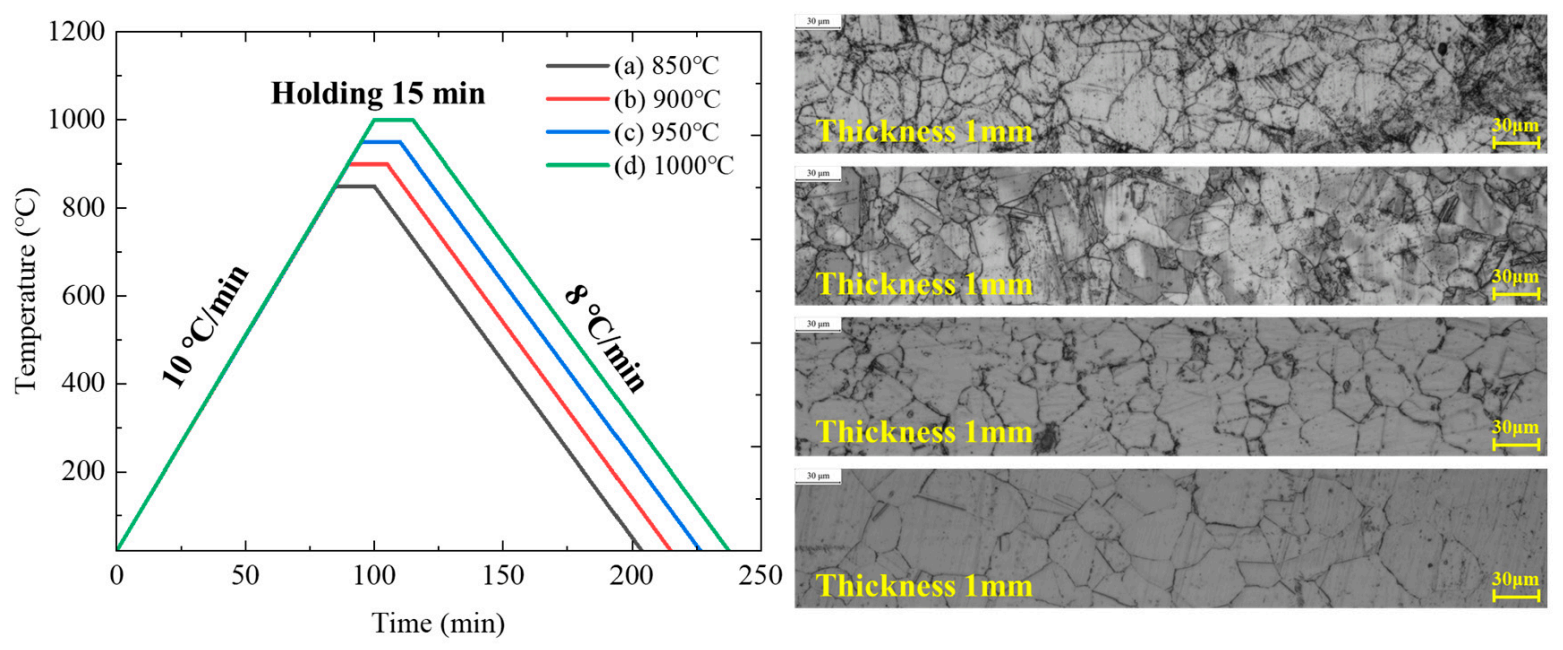
Microstructures of 304 SS sheets (0.1 mm) corresponding to different heat treatment temperatures.

Commercial 304 stainless steel (SS) thin sheets supplied by Zhongxi (Shandong) Metal Technology Co. Ltd. (Jinan, China) were used as the base material. The as-received 304 SS sheets were in cold-rolled annealed condition with a nominal thickness ranging from 0.05 mm to 0.15 mm. To obtain a range of grain sizes, heat treatments were conducted at four temperatures (850 °C, 900 °C, 950 °C, and 1000 °C) across five sheet thicknesses (0.05 mm, 0.08 mm, 0.10 mm, 0.12 mm, and 0.15 mm) using a miniature open-ended tube furnace. Prior to heat treatment, all sheets were ultrasonically cleaned in acetone for 15 min to remove surface contaminants and rolling oils. No mechanical pre-treatment (e.g., polishing or etching) was performed before heat treatment to avoid introducing surface deformation that could affect subsequent grain growth behavior. The corresponding microstructures of 304 SS sheets with a thickness of 0.10 mm after different heat treatment temperatures are presented in Figure 1. In detail, the specimens were heated from room temperature to the target temperature at a heating rate of 10 °C/min, followed by an isothermal holding period of 15 min. Subsequently, the specimens were furnace-cooled to room temperature at a controlled rate of 8 °C/min. Throughout the entire thermal cycle, a constant argon flow rate of 0.5 L/min was maintained. The average grain size was quantified using the linear intercept method in accordance with GB/T 6394-2017 [35]. For each specimen, statistical analysis was performed on three randomly selected fields of view at 500× magnification. The mean grain size was calculated using the equation *D* = *L*/(*N* × *M*), where *L* represents the total test line length, *N* is the number of intersections, and *M* denotes the magnification.

### 2.2. Uniaxial Tensile Tests

**Figure 2:**
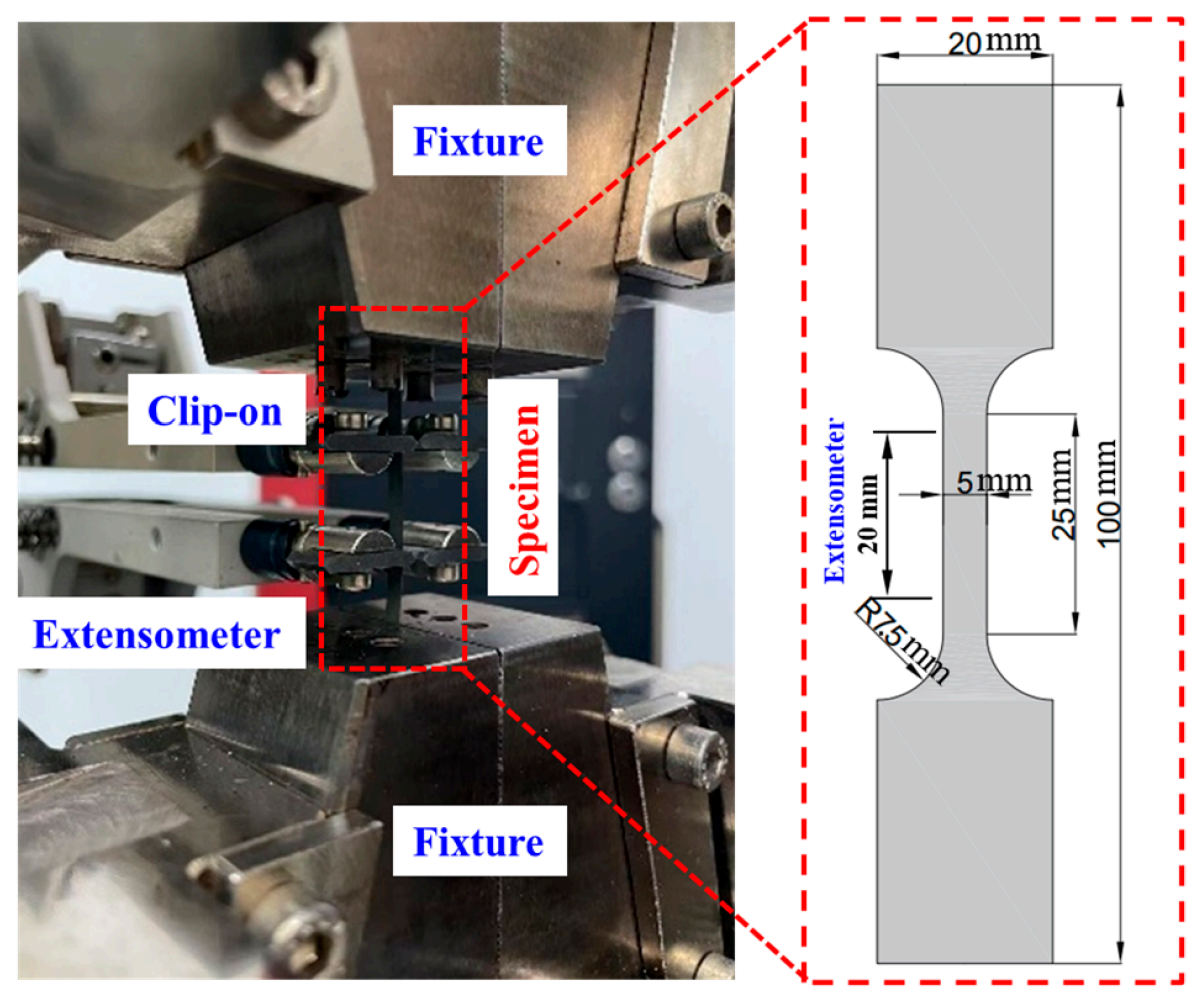
Experimental setup and specimen dimensions for uniaxial tensile tests.

Uniaxial tensile tests were conducted to characterize the mechanical behavior of 304 SS ultra-thin sheets under the coupled influence of grain coarsening and thickness reduction. Specifically, the dimensionless ratio *t*/*D*_m_ (sheet thickness to average grain size) was adopted as the governing parameter to quantify the relative contribution of surface grains versus interior grains to the overall mechanical response. By systematically varying the heat treatment temperature and selecting sheets of different initial thicknesses, a wide range of *t*/*D*_m_ ratios was achieved, enabling a comprehensive investigation of size effects across the transition from bulk-like to surface-dominated deformation. The tests were conducted following ASTM E8. The specimen geometry conformed to the standard’s thin-sheet configuration without dimensional adaptation, with a gauge width of 5 mm and a gauge length of 25 mm. The detailed dimensions and the experimental configuration are illustrated in Figure 2. The monotonic tensile tests were conducted on an MTS servo-hydraulic machine (5 kN) at ambient temperature. Tensile tests were performed at a crosshead displacement rate of 0.5 mm/min. With a gauge length of 25 mm, this corresponds to an initial engineering strain rate of approximately 3.3 × 10^−4^s^−1^. The clip-on extensometer had an initial gauge length of 20 mm, which is shorter than the specimen gauge length of 25 mm. The extensometer was carefully removed when the measured strain reached the maximum range of the extensometer (*ε* = 0.15). Beyond this point, the strain was determined from the crosshead displacement signal of the MTS testing machine through an iterative correction procedure. To accommodate the extremely thin specimens, a specialized clip-on extensometer was employed to ensure accurate strain measurement without inducing local stress concentrations. In addition, repeated tests (two specimens per condition) were conducted to ensure the reliability of experimental results. The surface flaws were assessed via visual inspection under 10× magnification using an optical microscope. To minimize experimental scatter arising from inherent material defects, only heat treatment specimens without scratches, pits and edge cracks were selected. Because the specimens were extremely thin, abrasive paper was bonded to the gripping surfaces to enhance friction and prevent slippage during loading. The abrasive paper was bonded exclusively to the gripping tabs to prevent slippage during loading. Since it did not extend into the gauge section, the measured mechanical response was unaffected.

## 3. Finite Element Modeling Incorporating Size Effects

### 3.1. Constitutive Model Incorporating Size Effects

Based on the framework, the composite model and the surface layer model, the flow stress is defined based on the variation of yield strength with the *t*/*D_m_* ratio. The composite model treats each grain as comprising a hard grain boundary and a soft interior. The single-grain flow stress *σ_s_*(*ε*) is as follows [36]:

(1) $$\sigma_{s}\left(\varepsilon \right)=\lambda \sigma_{i}\left(\varepsilon \right)+\left(1-\lambda \right)\sigma_{b}\left(\varepsilon \right)$$

where *σ_i_*(*ε*) and *σ_b_*(*ε*) are the grain interior and boundary flow stress, respectively. Unit: MPa. *λ* is the volume fraction of the grain interior.

Assuming the average grain size is *D_m_* (unit: mm) and the grain boundary layer thickness is *t_g_* (unit: mm), the relationship between them can be described by the following equation [36]:

(2) $$t_{g}=kD_{m}^{n}(0<n<1)$$

where *k* and *n* are material constants, defined as 0.133 and 0.7 [20], respectively. These values have been validated for 304 SS and similar FCC materials [20].

By substituting the grain with an equivalent sphere, where the grain size equals the diameter of the equivalent sphere, the volume of a single grain can be expressed as follows:

(3) $$V_{g}=\frac{4}{3}\pi {\left(\frac{D_{m}}{2}\right)}^{3}$$

(4) $$\lambda =\frac{\frac{4}{3}\pi {\left(\frac{D_{m}-t_{g}}{2}\right)}^{3}}{V_{g}}={\left(1-\frac{2t_{g}}{D_{m}}\right)}^{3}$$

The single-grain flow stress can then be expressed as follows

(5) $$\sigma_{s}\left(\varepsilon \right)={\left(1-\frac{2t_{g}}{D_{m}}\right)}^{3}\sigma_{i}\left(\varepsilon \right)+\left[1-{\left(1-\frac{2t_{g}}{D_{m}}\right)}^{3}\right]\sigma_{b}\left(\varepsilon \right)$$

Furthermore, the surface layer model is introduced. It assumes that the material comprises interior grains and surface grains. Accordingly, the macroscopic flow stress *σ*(*ε*) is divided into two components:

(6) $$\sigma \left(\varepsilon \right)=\eta \sigma_{inner}\left(\varepsilon \right)+\left(1-\eta \right)\sigma_{\mathrm{surf}}\left(\varepsilon \right)$$

where *σ_inner_*(*ε*) and *σ_surf_*(*ε*) are the interior and surface layer flow stresses, MPa. *η* is the volume fraction of interior grains.

The intragranular flow stress *σ_inner_*(*ε*) is given by *σ_s_*(*ε*), neglecting surface layer grain boundary strengthening. Assuming the surface layer flow stress *σ_surf_*(*ε*) equals the intragranular value *σ_i_*(*ε*), we obtain the following:

(7) $$\sigma \left(\varepsilon \right)=\eta \sigma_{s}\left(\varepsilon \right)+\left(1-\eta \right)\sigma_{i}\left(\varepsilon \right)=\left(1+\eta \lambda -\eta \right)\sigma_{i}\left(\varepsilon \right)+\eta (1-\lambda )\sigma_{b}\left(\varepsilon \right)$$

Given that the bipolar plate is a plate specimen, its overall volume is expressed as follows:

(8) $$V_{p}=w\times l\times t$$

where *w* is the width, *l* is the length, mm, and *t* is the plate thickness, mm.

By approximating the surface layer thickness as half the average grain size, and assuming the grains are uniformly distributed spheres, the number of grains within the surface layer can be calculated as follows:

(9) $$S_{g}=\pi {\left(\frac{D_{m}}{2}\right)}^{2}$$

(10) $$n_{1}=\frac{2a\times l}{S_{g}}$$

(11) $$n_{2}=\frac{2a\times t}{S_{g}}$$

(12) $$n_{3}=\frac{2l\times t}{S_{g}}$$

(13) $$n_{b}=n_{1}+n_{2}+n_{3}$$

where *n*_1_, *n*_2_ and *n*_3_ represent the approximate number of grains on the three types of surfaces of the bipolar plate (i.e., the top and bottom, left and right, and front and back surfaces), respectively, and *n_b_* is the total number of grains in the surface layer of the bipolar plate.

The number of grains in the interior of the material is given by the following:

(14) $$n_{g}=\frac{V_{g}}{V_{i}}$$

(15) $$V_{i}=\left(a-D_{m}\right)\left(l-D_{m}\right)\left(t-D_{m}\right)$$

where *n_g_* is the number of grains in the interior of the bipolar plate material, and *V_i_* is the volume of the material after excluding the surface layer, mm^3^.

Based on the number of grains, the volume fraction of interior grains in the bipolar plate material can be expressed as follows:

(16) $$\eta =\frac{n_{g}}{n_{b}+n_{g}}$$

Substituting Equations (4) and (16) into Equation (7) yields the following:

(17) $$\sigma \left(\varepsilon \right)=\left\{1+\eta \left[{\left(1-\frac{2t_{g}}{D_{m}}\right)}^{3}-1\right]\right\}\sigma_{i}\left(\varepsilon \right)+\eta \left[1-{\left(1-\frac{2t_{g}}{D_{m}}\right)}^{3}\right]\sigma_{b}\left(\varepsilon \right)$$

Substituting Equations (2) and (3) into Equations (3)–(17), the flow stress of the bipolar plate material can be expressed as follows:

(18) $$\sigma \left(\varepsilon \right)=\left\{1+\eta \left[{\left(1-0.266D_{m}^{-0.3}\right)}^{3}-1\right]\right\}\sigma_{i}\left(\varepsilon \right)+\eta \left[1-{\left(1-0.266D_{m}^{-0.3}\right)}^{3}\right]\sigma_{b}\left(\varepsilon \right)$$

To comprehensively reveal the effects of grain size and sheet thickness on the internal stress, it is necessary to decouple and quantify two independent stress response mechanisms based on experimental data:

(1) Grain-size-dominated: Constant thickness *t* with varying average grain size *D_m_*.
(2) Thickness-dominated: Constant grain size *D_m_* with varying thickness *t*.

Taking the first case as an example, the flow stress functions *σ_i_*(*ε*) and *σ_b_*(*ε*) corresponding solely to grain size variation can be inversely identified by solving Equation (18) simultaneously. This yields the grain-size-dominated flow stress, denoted as *σ_Dm_*(*ε*) (obtained by fitting with fixed thickness and varying grain size). Similarly, the thickness-dominated flow stress, denoted as *σ_t_*(*ε*) (obtained by fitting with fixed grain size and varying thickness), can be derived from the second case.

Based on Refs. [19,36], at the micro/meso-scale, the yield strength increases approximately linearly with the ratio of thickness to grain size (*t*/*D_m_*). Accordingly, a linear weighted superposition strategy is introduced to construct the final comprehensive constitutive relation by integrating the two types of flow stresses after size-effect correction:

(19) $$\sigma \left(\varepsilon \right)=\left(\gamma_{Dm}\sigma_{D_{m}}\left(\varepsilon \right)+\gamma_{t_{m}}\sigma_{t}\left(\varepsilon \right)\right)/2$$

where 1/2 is the normalization constant, *γ_Dm_* + *γ_t_* = 2. *γ_Dm_* and *γ_t_* represent the influence factors of size effects induced by grain size and sheet thickness variations, respectively, defined as follows:

(20) $$\gamma_{D_{m}}=1+\frac{\sigma_{y}\left(D_{0},t_{0}\right)-\sigma_{Dm}(D_{0},t_{0})}{\sigma_{Dm}(D_{0},t_{0})}$$

(21) $$\gamma_{t_{m}}=1+\frac{\sigma_{y}\left(D_{0},t_{0}\right)-\sigma_{tm}(D_{0},t_{0})}{\sigma_{tm}(D_{0},t_{0})}$$

here, *t*_0_ and *D*_0_ are the target thickness and average grain size of the bipolar plate material, respectively; *σ_Dm_*(*D*_0_*,t*_0_) and *σ_t_*(*D*_0_*,t*_0_) are the yield strengths calculated from the *σ_Dm_*(*ε*) and *σ_t_*(*ε*) curves, respectively; and *σ_y_*(*D*_0_*,t*_0_) is the experimentally measured yield strength of the target material.

### 3.2. Finite Element Geometric Modeling

Given the significantly high width-to-thickness ratio of the sheet metal, the stress component normal to the sheet surface is negligible, thereby validating the plane-stress assumption. Consequently, a two-dimensional geometric model was employed to efficiently resolve the transverse deformation mechanics, specifically focusing on through-thickness stress gradients. It is important to note that while this 2D simplification provides a robust framework for analyzing cross-sectional formability, it does not account for in-plane anisotropy or complex 3D edge effects, which are considered secondary factors in the context of the current investigation. A two-dimensional geometric model incorporating grain/grain boundary structures is essential, which characterizes the stress–strain response and microstructural evolution during stamping. In this paper, a representative volume element model (2D polycrystalline model) was established to verify the accuracy of the proposed constitutive model and a three-channel stamping finite element model was proposed to investigate the formability of ultra-thin metal bipolar plates.

**Figure 3:**
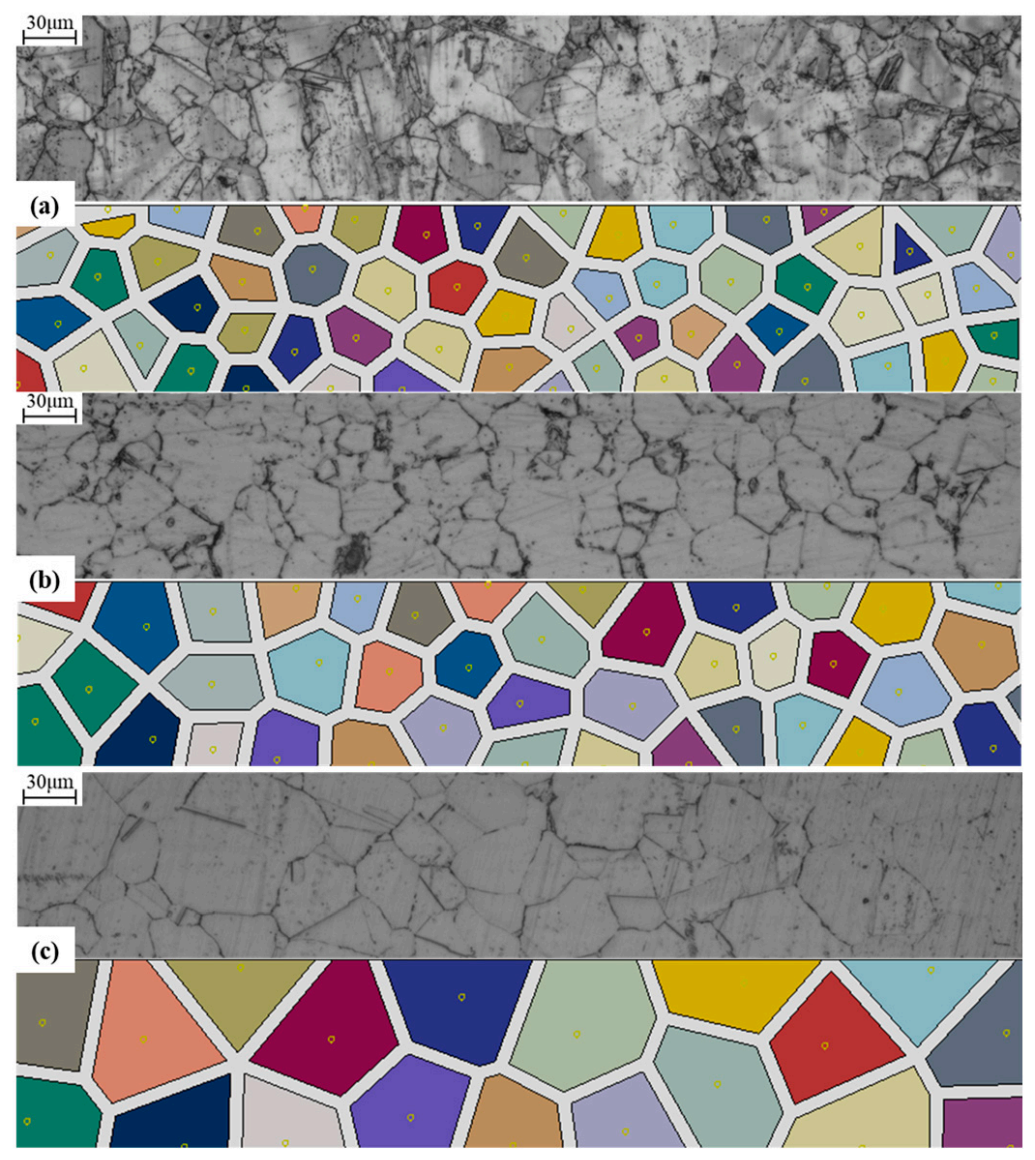
Comparison between metallographic structures and Voronoi-generated microstructures for 304 SS with different average grain sizes: (**a**) 24 μm, (**b**) 30 μm and (**c**) 51 μm.

A 2D polycrystalline model for 304 stainless steel was constructed via Voronoi tessellation. First, the number of Voronoi seeds was quantitatively determined based on the measured average grain diameter (*D_m_*) and the required model dimensions. These seeds were then spatially distributed using a random function (rand) before the Voronoi algorithm was applied to partition the grain regions. To explicitly model the grain boundaries, a geometric offset operation was applied to the Voronoi edges based on *D_m_*. Figure 3 validates the model’s fidelity by comparing the generated Voronoi morphologies with experimental metallographic microstructures for *D_m_* = 24, 30 and 51 μm (white: grain boundaries; colored: grain interiors). To account for the stochastic nature of the Voronoi tessellation, five independent random realizations were generated for each grain-size condition.

**Figure 4:**
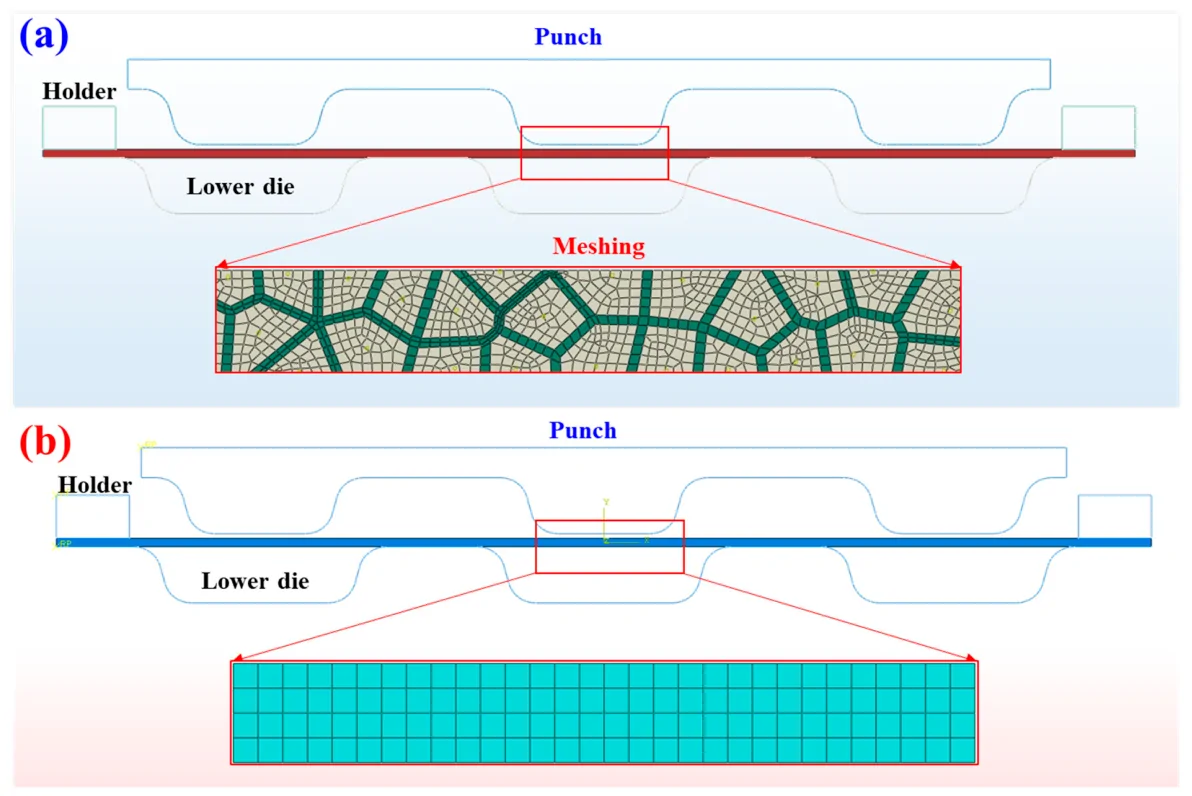
Finite element models for bipolar plate stamping: (**a**) polycrystalline model with explicit grain structure; (**b**) homogenized continuum model.

Based on this, a 2D three-channel stamping finite element model was established (Figure 4a). The sheet thickness is 0.1 mm with an average grain size of 0.05 mm. The geometric parameters of the flow channel are: width *W* = 1.5 mm, draft angle *α* = 15°, corner radius *R* = 0.3 mm, channel depth *h* = 0.65 mm, and rib width *S* = 1 mm. Given that the hardness of the punch, die, and blank holder significantly exceeds that of the sheet metal, these stamping components were modeled as analytical rigid bodies, with an isotropic point mass inertia of 0.05 t assigned to the punch. The sheet was discretized using the explicit plane stress elements (CPS4R) with a density of 7.93 g/cm^3^. Distinct constitutive parameters for the grain interiors and grain boundaries were implemented via user-defined subroutines. All interactions in the model were defined using surface-to-surface contact with a penalty friction formulation (friction coefficient of 0.1 and finite sliding algorithm). A mesh convergence study was conducted by simulating the stamping process with five element sizes: 5 μm, 9.3 μm, 14.7 μm, 19.2 μm and 25 μm (characteristic element edge length in the deformation zone). The maximum von Mises stress and the minimum thickness were compared across the five cases. The difference between the two finest meshes was less than 5.7%, confirming mesh convergence. The workpiece was meshed using quadrilateral elements with an approximate global size of 9.3 μm. Since the punch, die, and blank holder were modeled as analytical rigid bodies, they were meshed with a finer approximate global size of 1 μm. The die and blank holder were fully fixed. The punch was displaced at a constant speed of 0.065 mm/s for 10 s, followed by an unloading displacement of 0.5 mm.

For comparison, a homogenized model without grain microstructures is presented in Figure 4b. While the homogenized continuum model is effective for predicting the macroscopic stress field, it inherently averages out local material heterogeneity. To overcome this limitation, the polycrystalline model with explicit grain structure was established. Unlike the continuum approach, this model explicitly resolves the stress evolution within grains and at grain boundaries, thereby providing a detailed understanding of the local deformation mechanisms that govern macroscopic formability.

## 4. Results and Discussion

### 4.1. Experimental Results

The measured grain sizes are summarized in Table 1. Taking the 0.10 mm sheet as an example, the average grain size (d) increased from 20 ± 1 μm at 850 °C to 24 ± 2 μm at 900 °C and 30 ± 2 μm at 950 °C, with the most pronounced coarsening occurring between 950 °C and 1000 °C (an increase of 21 μm). This temperature-driven trend was consistent across all thicknesses. The influence of sheet thickness, however, varied with heat-treatment temperature: at 850–950 °C, increasing the thickness from 0.05 mm to 0.15 mm raised d by 19–24 μm, indicating a pronounced thickness dependence in this range, whereas at 1000 °C the corresponding increase was only 7 μm. These results indicate that both temperature and sheet thickness affect grain growth within the investigated range; thickness exerts a more prominent influence at lower annealing temperatures, whereas temperature-driven growth dominates at 1000 °C, thereby reducing the relative contribution of thickness. The reported error bars (±) correspond to one standard deviation derived from nine independent measurements.

**Figure 5:**
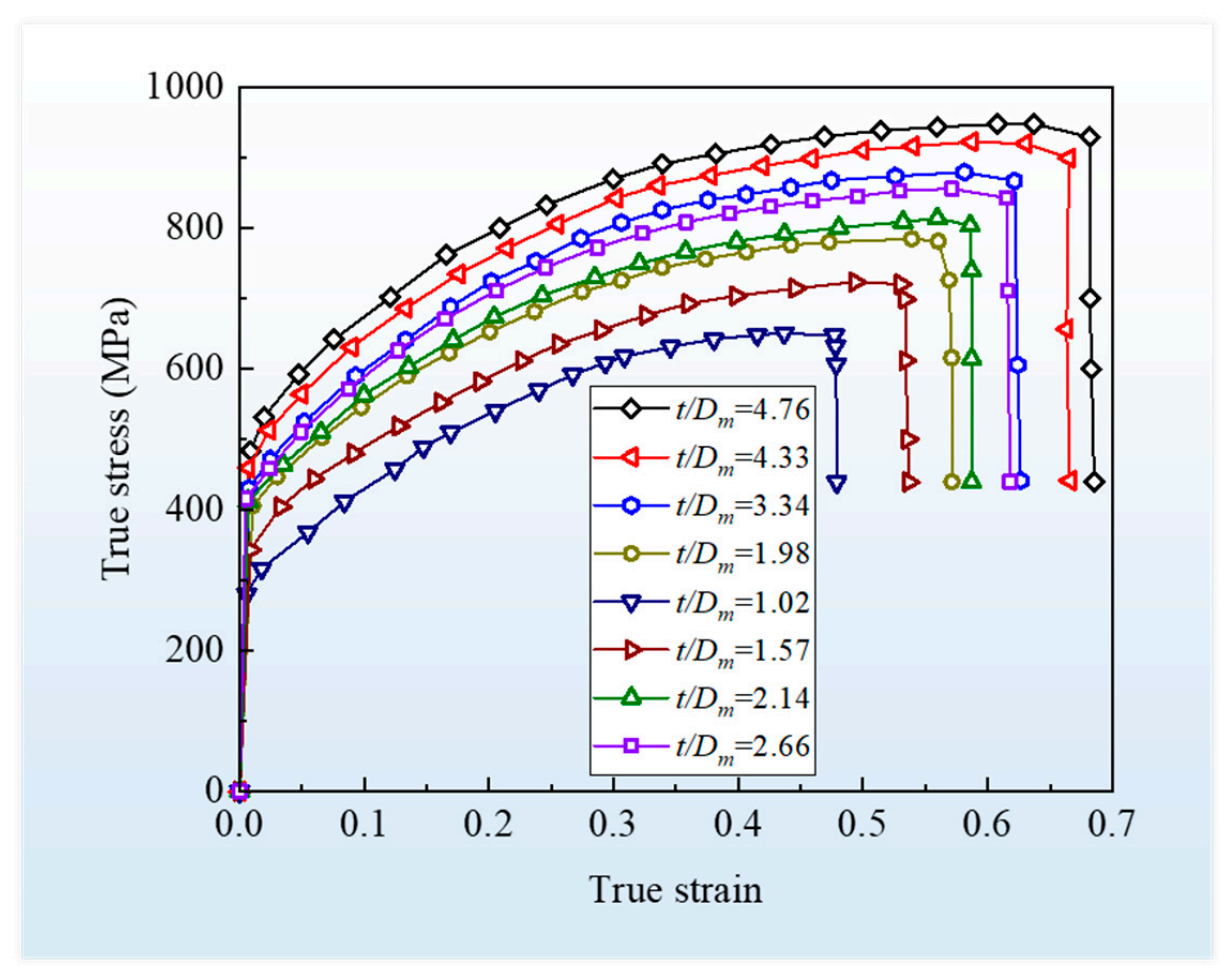
True stress–true strain curves for specimens with different thickness-to-average-grain-size ratios.

The ratio of sheet thickness to average grain size (*t*/*D_m_*) was adopted to characterize the number of through-thickness grain layers. As shown in Figure 5, flow stress increases monotonically with *t*/*D_m_*, i.e., yield strength rises from 280 MPa (*t*/*D_m_* = 1.02) to 483 MPa (*t*/*D_m_* = 4.76), ultimate tensile strength from 650 MPa to 948 MPa, and true strain from 0.48 to 0.68. Mechanistically, a higher *t*/*D_m_* ratio indicates a greater number of through-thickness grain layers, resulting in a higher overall grain boundary density. Since grain boundaries act as obstacles to dislocation motion and promote dislocation pile-up, the increased boundary density enhances the resistance to plastic deformation, which manifests macroscopically as elevated flow stress [37].

### 4.2. Validation of the Constitutive Models

To establish a constitutive model for bipolar plates that accounts for the coupled size effects of grain size and sheet thickness, the two unknown component flow stresses in Equation (18), *σ_i_*(*ε*) and *σ_b_*(*ε*), must first be determined. Based on the uniaxial tensile test results presented in Section 2.2, true stress–strain curves were obtained for four groups of 304 stainless steel sheet specimens with the following parameters.

Step 1: Acquisition of experimental data. Based on the uniaxial tensile tests described in Section 2.2, true stress–strain curves were obtained for four groups of 304 stainless steel specimens, covering two sheet thicknesses (*t* = 0.05 mm and 0.15 mm) and two average grain sizes (*D*_m_ corresponding to heat treatment temperatures of 850 °C and 1000 °C).

Step 2: Identification of *σ*_Dm_(*ε*) and *σ_t_*(*ε*). For each of the four specimen groups, the measured thickness *t*, average grain size *D*_m_, and experimental stress–strain data *σ*(*ε*) were substituted into Equation (18). This yielded a system of equations from which the grain-size-dominated flow stress *σ*_Dm_(*ε*) and the thickness-dominated flow stress *σ*_t_(*ε*) were derived. Specifically, *σ*_Dm_(*ε*) captures the contribution of grain size to the flow stress, while *σ*_t_(*ε*) captures the contribution of sheet thickness.

Step 3: Relationship between *σ_i_*(*ε*), *σ_b_*(*ε*), *σ*_Dm_(*ε*), and *σ_t_*(*ε*). It should be clarified that *σ*_Dm_(*ε*) and *σ*_t_(*ε*) are not independent of *σ*_i_(*ε*) and *σ*_b_(*ε*). According to Equation (18), *σ*_Dm_(ε) and *σ_t_*(*ε*) are derived from *σ_i_*(*ε*) and *σ_b_*(*ε*) through the grain-size- and thickness-dependent weighting factors, respectively.

Once *σ*_Dm_(*ε*) and *σ_t_*(*ε*) are determined from Step 2, the underlying *σ*_i_(*ε*) and *σ*_b_(*ε*) can be back-calculated if needed, or the model can proceed directly to the superposition step.

Step 4: Prediction for a target specimen (example: *t* = 0.12 mm). For a target specimen with *t* = 0.12 mm and its corresponding average grain size *D*_m_:

(a) The values of t and *D*m are substituted into the previously derived *σ*_Dm_(*ε*) and *σ_t_*(*ε*) functions to obtain the grain-size-dominated and thickness-dominated true stress–strain curves, respectively.
(b) The yield strengths *σ*_Dm_(*D*_0_,*t*_0_) and *σ_t_*(*D*_0_,*t*_0_) are evaluated at the initial conditions, and together with the experimentally measured overall yield strength *σ_y_*(*D*_0_,*t*_0_), the weighting coefficients in Equation (19) are determined.
(c) The final equivalent true stress–strain relationship is synthesized via the weighted superposition defined in Equation (19).

**Figure 6:**
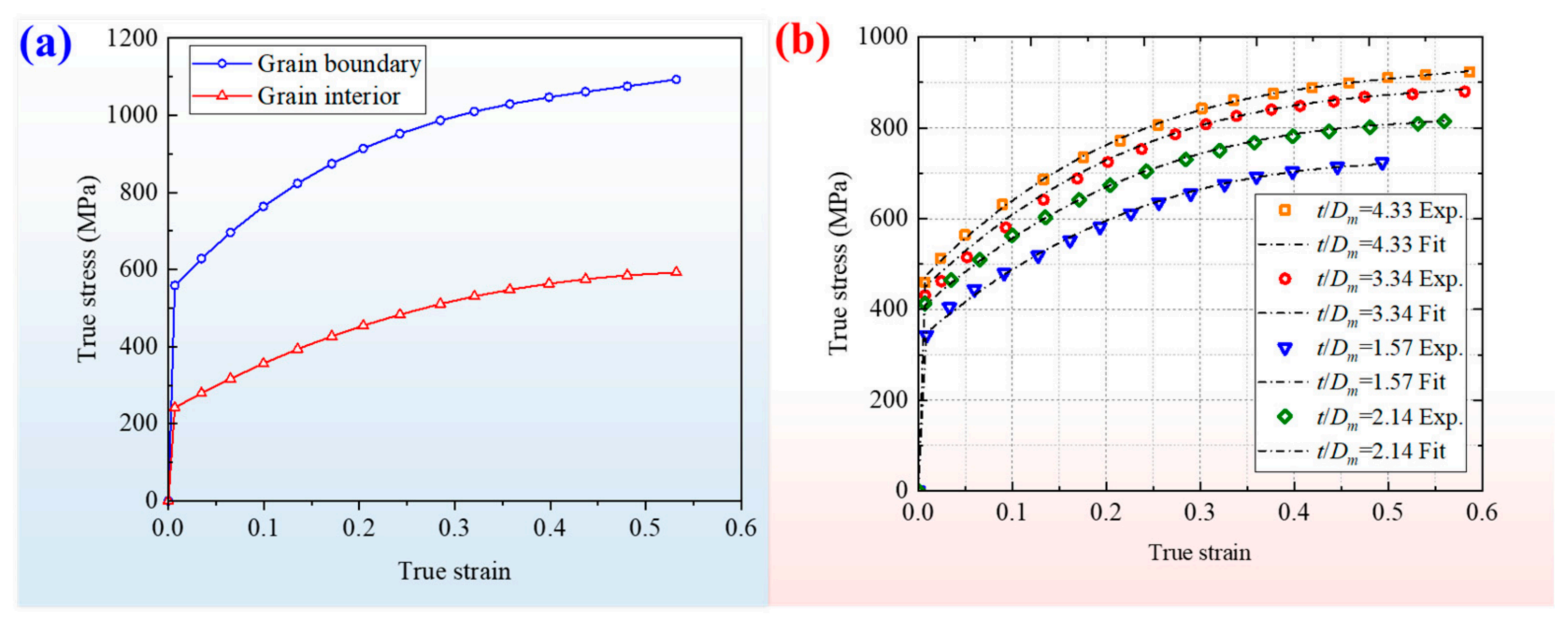
Validation of the constitutive model: (**a**) true stress–true strain curves of grain boundary and grain interior; (**b**) comparison between predicted and experimental macroscopic curves for various *t*/*D_m_* ratios.

Figure 6a shows that the stress differential between grain boundaries and interiors widens with strain, attributed to dislocation pile-up and higher boundary dislocation density. This result is in close accordance with that reported in Ref. [37]. Following the same procedure, the overall macroscopic stress–strain curves as well as the individual responses of grain boundaries and grain interiors were solved for various combinations of sheet thickness and average grain size (*t*/*d* = 4.33, 3.34, 1.57 and 2.14). As presented in Figure 6b, the fitted curves align closely with the experimental points, which were not used for parameter fitting but reserved for independent validation. The maximum deviation along the data line of 21.26 MPa occurs at *t*/*D_m_* = 3.34, with a relative error of 4.6%. The relative error is defined as the difference between the FE-predicted results and the experimental measurements, normalized by the corresponding experimental values. These results demonstrate that the proposed constitutive model can accurately characterize the true stress–strain behavior of ultra-thin bipolar plate materials.

**Figure 7:**
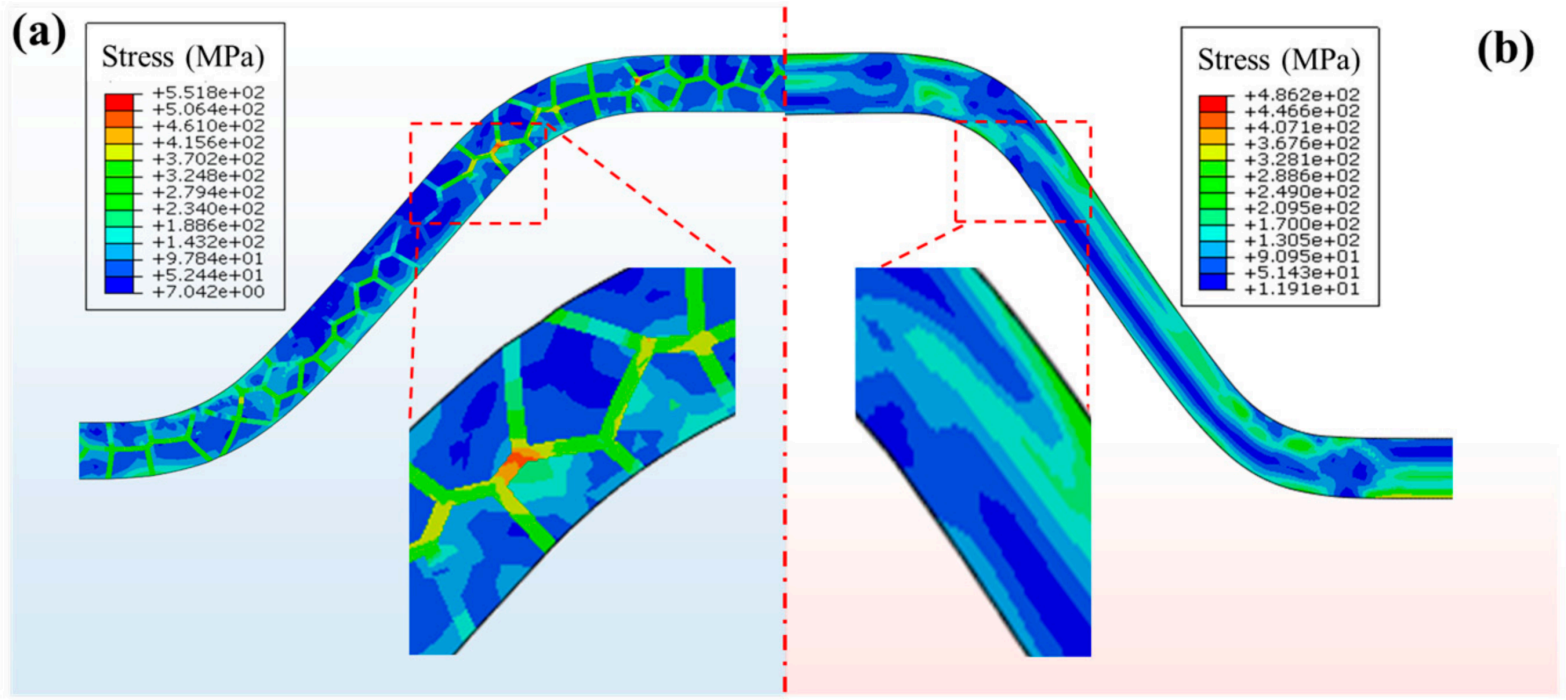
Comparison of stress distributions between (**a**) the model considering grain microstructures and (**b**) the homogenized model without grain microstructures.

The models with explicit resolved grain microstructures exhibit a markedly different stress distribution from that of the homogenized model, as shown in Figure 7. In the homogeneous model, the stress field is relatively uniform, with no notable local stress concentration, and the peak stress reaches 486.2 MPa at the channel fillet. In contrast, the model incorporating grain boundaries shows clear stress localization at boundary regions, raising the maximum von Mises stress to 551.8 MPa. Under the same loading conditions, neglecting grain-scale heterogeneity inevitably introduces prediction deviations, because the homogenized approach fails to capture the dislocation-pinning effect of grain boundaries and the associated local stress concentrations. While this numerical contrast demonstrates that the homogenized model cannot capture grain-scale stress localization, the definitive conclusion that explicit grain structures are ‘necessary’ requires experimental validation. Such validation is beyond the scope of the present study. However, the grain-resolved model is expected to provide more physically realistic predictions because the stress concentrations at grain boundaries are known to influence deformation mechanisms in ultra-thin sheets.

### 4.3. Effects of Grain Size and Plate Thickness on Stress Behavior

**Figure 8:**
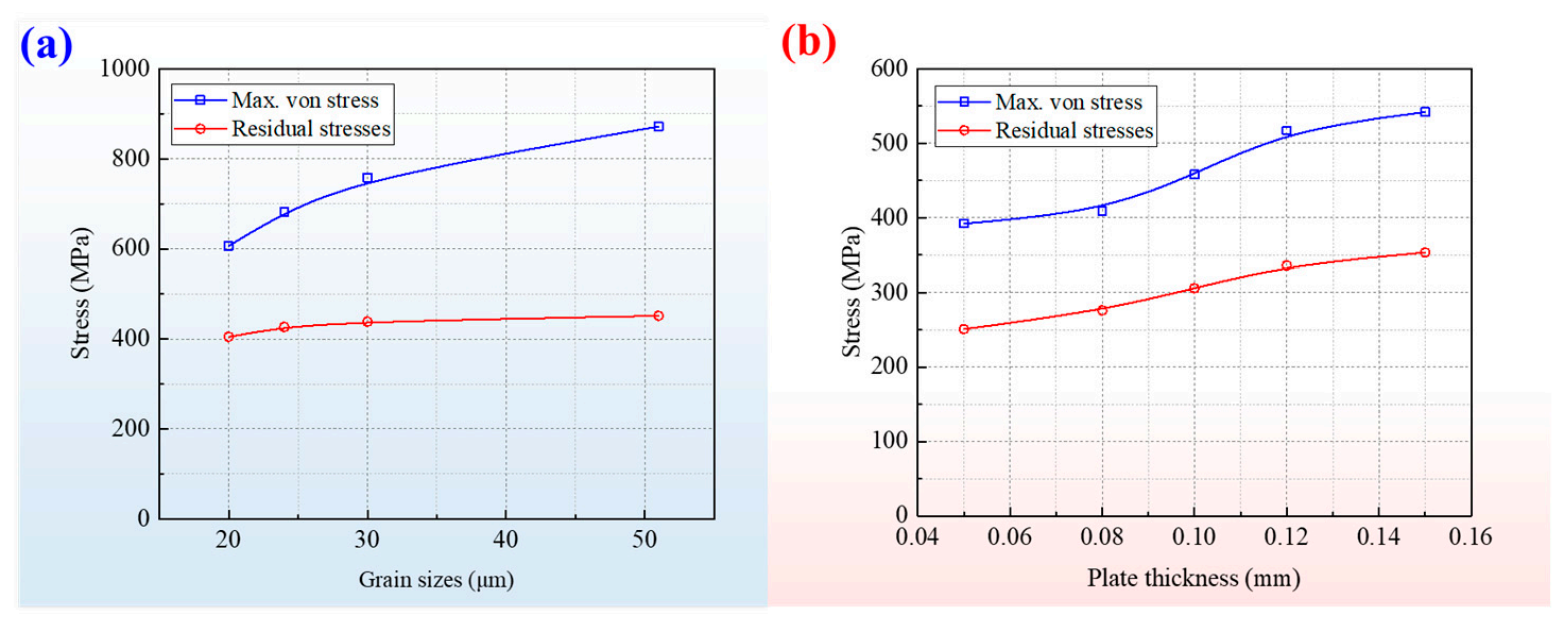
Effects of (**a**) grain size and (**b**) plate thickness on the maximum von Mises stress during stamping and after unloading.

The effects of grain size and plate thickness on the maximum von Mises stress are illustrated in Figure 8a,b, respectively. During the stamping stage, the maximum von Mises stress increases monotonically with grain size. For average grain sizes of 20 μm, 24 μm, 30 μm, and 51 μm, the corresponding maximum stresses are 606 MPa, 681 MPa, 758 MPa, and 872 MPa, respectively, representing relative increases of 12.4%, 25.1%, and 44.4% with respect to the 20 μm baseline. After unloading, the residual stresses are 405 MPa, 427 MPa, 439 MPa, and 452 MPa, respectively, with smaller relative increases of 5.4%, 8.4%, and 11.6%, respectively. These results indicate that an increase in the average grain size leads to a significant intensification of the local stress concentration at the fillet. This phenomenon can be attributed to the fact that, at a constant sheet thickness, a larger grain size reduces the number of grains across the thickness direction, thereby increasing deformation inhomogeneity. Consequently, the coarser grains are more prone to severe strain localization at the fillet, resulting in elevated peak and residual stress levels.

As shown in Figure 8b, the influence of plate thickness on the maximum von Mises stress follows an identical trend to that of grain size. As the plate thickness increases from 0.05 mm to 0.15 mm, the maximum von Mises stress rises from 394.6 MPa to 545.1 MPa, and the residual stress increases from 251.4 MPa to 352.0 MPa. These results imply that, within the investigated range, finer grains and reduced plate thickness both help in lowering forming and residual stresses. A lower stress level is advantageous for reducing the risk of damage in metallic bipolar plates during elevated temperature operation.

### 4.4. Effects of Grain Size and Plate Thickness on Thinning Rate

**Figure 9:**
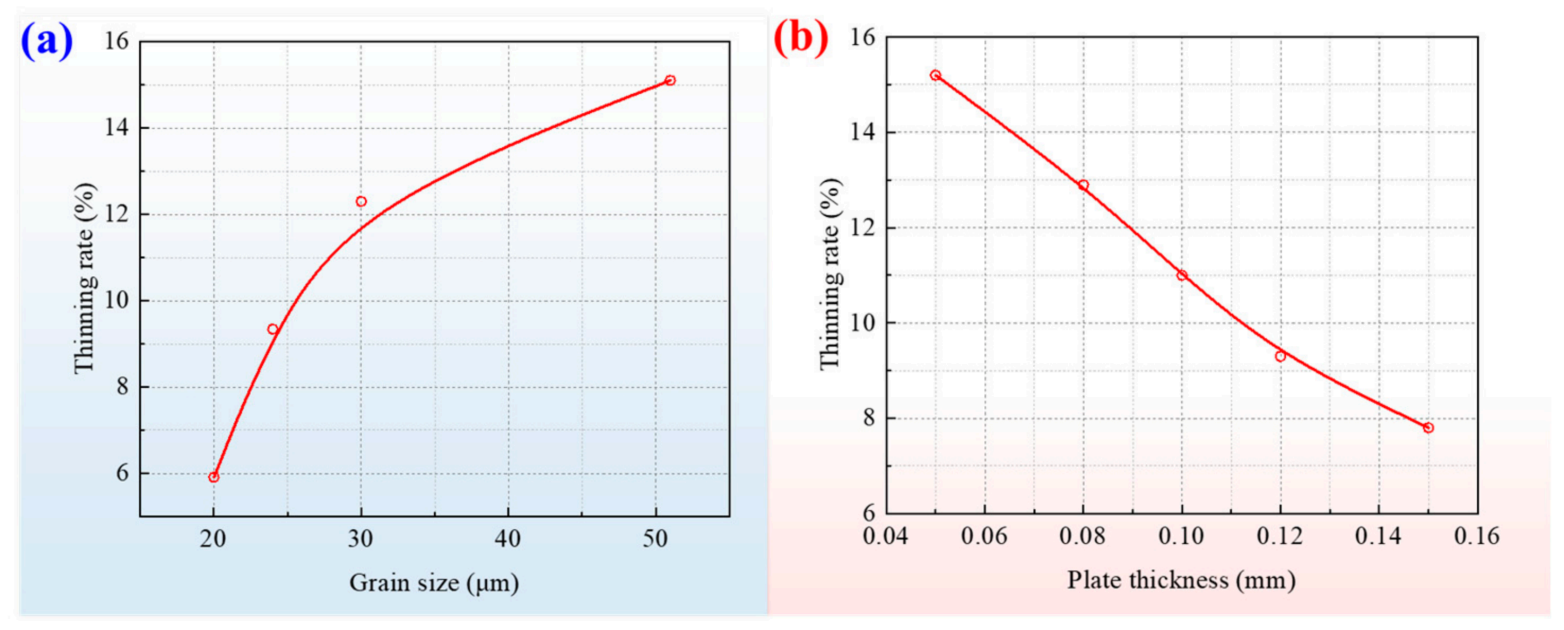
Effects of (**a**) grain size and (**b**) plate thickness on the maximum thinning rate.

The effects of grain size and sheet thickness on the maximum thinning rate are illustrated in Figure 9a,b, respectively. Thinning rate = (initial thickness- final thickness)/initial thickness × 100%. During stamping, the maximum thinning rate increases monotonically with grain size. For average grain sizes of 20 μm, 24 μm, 30 μm, and 51 μm (thickness 0.1 mm), the corresponding thinning rates are 5.91%, 9.34%, 14.3%, and 15.1%, respectively. This indicates that resistance to localized thinning deteriorates significantly as grain size increases. Mechanistically, for a given plate thickness, a smaller average grain size yields a higher density of grain boundaries, allowing the plastic deformation to be distributed over a larger number of grains. This promotes more homogeneous plastic flow and suppresses strain localization. Conversely, coarse grains concentrate plastic strain into fewer grains, accelerating local thickness reduction.

As shown in Figure 9b, the plate thickness affects thinning in an opposite manner to grain size. As the plate thickness increases from 0.05 mm to 0.15 mm (gain size 51 μm), the maximum thinning rate decreases from 15.2% to 7.8%. Thicker sheets exhibit stronger resistance to thickness reduction due to enhanced through-thickness constraints and altered stress states. In ultra-thin sheets, the weak lateral constraints imposed by free surfaces facilitate dislocation escape, reduce strain hardening capacity, and promote a plane-stress state dominated by thinning. With increasing thickness, multiple grain layers develop through the thickness direction, giving rise to a more triaxial stress state in which through-thickness compressive stresses help resist further thinning. Furthermore, the increased material volume facilitates redistribution of local plastic strain: hard grains constrain excessive deformation of neighboring soft grains, promoting lateral strain spreading rather than shear band localization. Collectively, these mechanisms enable thicker sheets to achieve more homogeneous through-thickness deformation, delayed necking onset, and a significantly reduced maximum thinning rate. The simulation results directly attribute the reduced thinning in thicker samples to the intensified through-thickness constraints and the consequent stress-state transition. While microscopic mechanisms such as dislocation escape and shear-band suppression were not explicitly modeled, the observed macroscopic stability aligns with these established theories, suggesting they act as the underlying physical basis for the delayed necking observed in our continuum model.

### 4.5. Effects of Grain Size and Plate Thickness on Springback

**Figure 10:**
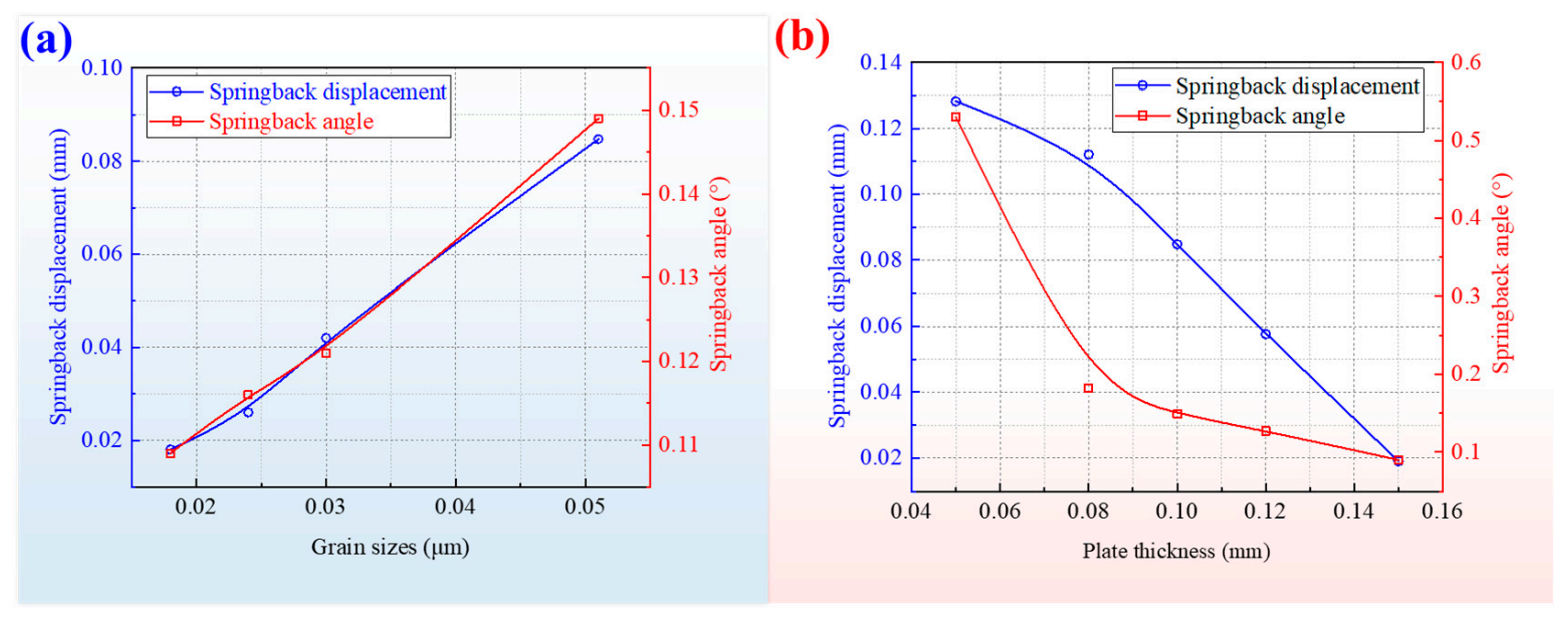
Effects of (**a**) grain size and (**b**) plate thickness on springback displacement and springback angle.

The effects of grain size on springback displacement and springback angle are illustrated in Figure 10a. A node set is defined by selecting the right endpoint of the upper surface of the left rib, and the left and right endpoints of the intermediate channel. The springback angle is determined by the difference in the angle of this node set before and after unloading. The springback displacement is defined as the displacement of the intermediate channel along the *y*-axis before and after unloading. Both metrics increase with increasing grain size: as the average grain size increases from 20 μm to 50 μm, the springback displacement rises from 0.018 mm to 0.083 mm, and the springback angle increases from 0.1° to 0.149°. During stamping, grain boundaries serve as obstacles to dislocation motion. Sheets with larger grain sizes possess a lower overall grain boundary density, which reduces resistance to plastic flow and results in a greater proportion of elastic strain accumulation at a given load level. Upon unloading, the higher stored elastic strain energy drives more pronounced springback. Furthermore, in coarse-grained sheets, intragranular dislocation slip accommodates a larger fraction of the total deformation, facilitating more complete elastic strain release and thereby amplifying the springback response. The above mechanistic interpretation is adopted from Ref. [37] and is not directly derived from the present simulations. Figure 10b presents the calculated springback displacement and springback angle as functions of plate thickness. As the sheet thickness increases from 0.05 mm to 0.15 mm, both the springback displacement and springback angle decrease monotonically from 0.124 mm to 0.020 mm and from 0.54° to 0.09°, respectively. This trend is primarily attributed to the microstructural evolution in ultra-thin sheets. The increase in springback with decreasing sheet thickness can be attributed to three factors: (i) thinner sheets have lower bending rigidity (∝t^3^), resulting in a larger elastic fraction of the total deformation; (ii) the higher surface-grain fraction in thinner sheets reduces the flow stress, thereby increasing the elastic-to-total strain ratio; and (iii) the resulting through-thickness stress heterogeneity amplifies the bending moment driving elastic recovery. It should be noted that a quantitative decomposition of these contributions is beyond the scope of the present work.

## 5. Optimization of Die Parameters for Bipolar Plates

In addition to grain size and plate thickness, die geometric parameters play a critical role in determining the forming quality of bipolar plates. In high-precision stamping, geometric parameters such as draft angle (*α*), fillet radius (*R*), channel depth (*h*), channel width (*W*) and rib width (*S*) are nonlinearly coupled with forming-quality indicators, including thinning rate and springback angle. To address this complexity, an intelligent optimization framework integrating a machine-learning-based surrogate model with a global heuristic optimization algorithm was developed.

### 5.1. Data Preprocessing and Regression Models

During data preprocessing, 41 sets of structured simulation data, summarized in Table 2, were first imported using the Pandas library. To eliminate numerical biases arising from differences in parameter scales and units, the StandardScaler method was applied to standardize all input features, transforming them into a distribution with zero mean and unit variance.

Five critical process parameters were selected as the input feature matrix **X**. The thinning rate and springback angle were designated as the output response matrix **Y**. The complete dataset was then randomly partitioned into training and testing sets according to a predefined ratio. This partitioning strategy was used to evaluate the model’s generalization ability on unseen data while reducing the risk of overfitting caused by memorization of training samples.

To achieve high-accuracy response prediction within seconds across a multidimensional process-parameter space, machine-learning-based surrogate models were used in place of computationally expensive conventional FEM simulations in this study. Specifically, Random Forest (RF) and XGBoost regression algorithms were employed to construct high-fidelity surrogate models. To eliminate potential interference between output variables during the simultaneous prediction of thinning rate and springback angle, the MultiOutputRegressor framework was adopted to decouple the multi-output prediction task. This strategy enables each response variable to be modeled independently, thereby improving both the stability and reliability of the predictive models.

### 5.2. Analysis and Optimization Results

After the surrogate models were trained and their predictive accuracy verified on the test set, the optimization stage was initiated. The objective was to identify the optimal parameter configuration within the physically feasible five-dimensional space that simultaneously minimizes the thinning rate and springback angle.

The Differential Evolution (DE) algorithm was specifically selected to overcome the gradient-vanishing trap inherent in tree-based surrogate models. Because the multidimensional response surfaces constructed by Random Forest and XGBoost are microscopically discrete, exhibiting step-like or flat landscapes, their local gradients are typically zero. Conventional gradient-based optimizers, such as SLSQP, are prone to premature convergence and entrapment in local optima within these flat regions. To address this, the differential_evolution function from the SciPy Optimize library was employed. As a gradient-free, population-based global heuristic, DE initializes a random population within the bounded parameter space and drives evolution through mutation, crossover, and selection operations based on inter-individual differences.

To accommodate the objectives within a single-objective DE framework, a composite fitness function was formulated to transform the multi-objective forming requirements into a scalar minimization task:

(22) $$Objective=\chi_{1}\hat{\delta }+\chi_{1}\hat{f}$$

where + denotes co-directional optimization, *δ* and *f* are the thinning rate and springback angle, and *χ*_1_ and *χ*_2_ are the weight coefficients (*χ*_1_ = *χ*_2_ = 0.5), respectively.

Through successive population evolution, the DE algorithm automatically searches the entire design space to identify the process-parameter combination that achieves concurrent minimization of the thinning rate (*δ*) and maximization of the springback angle (*f*) (Table 3).

According to the validation results (Table 4), both surrogate models exhibited high predictive accuracy on previously unseen data. In particular, the mean absolute errors associated with the predicted springback angle (*f*) were maintained at very low levels, confirming the reliability of the surrogate-assisted optimization. In terms of global optimization, the RF-DE framework demonstrated superior engineering applicability. Finally, the related finite element simulations were conducted using two different die sizes to evaluate their relative superiority. The results are summarized in Table 5. The non-standard parameter combination identified by this framework (*α* = 16.0°, *R* = 0.30 mm, *h* = 0.48 mm, *W* = 1.46 mm, *S* = 0.73 mm) successfully reduced the predicted thinning rate to 4.43% while simultaneously achieving a predicted springback angle of 0.151°. Such decimal-precision parameter configurations represent a solution space that is inaccessible to traditional orthogonal experimental design methods.

## 6. Conclusions

This study systematically investigated the effects of grain size and sheet thickness on the formability in micro-stamping of ultra-thin 304 stainless steel sheets. Random Forest (RF) and XGBoost surrogate models coupled with Differential Evolution (DE) were employed to optimize key stamping die parameters. The main conclusions are summarized as follows:

(1) Increasing the grain size from 20 μm to 51 μm raises the maximum von Mises stress, thinning rate, and springback angle, indicating a clear deterioration in formability. These deteriorations are attributed to reduced grain boundary density, which promotes stress concentration, localizes plastic strain within coarse grains, and increases stored elastic energy.
(2) Increasing the plate thickness from 0.05 mm to 0.15 mm increases the maximum von Mises stress due to enhanced triaxial constraint, but suppresses both the thinning rate and the springback angle, benefiting from improved strain redistribution and plastic dissipation capacity.
(3) Grain coarsening consistently degrades formability by simultaneously increasing stress, thinning and springback, whereas plate thickening introduces a trade-off between higher forming stress and improved dimensional stability. Therefore, grain refinement and appropriate thickness selection should be considered together in ultra-thin sheet stamping.
(4) The RF-DE framework identified an optimal non-standard parameter combination that achieves a predicted thinning rate of 4.43% and a springback angle of 0.151°. This shows the capability of surrogate-assisted global optimization to access a parameter space beyond the resolution of conventional orthogonal experimental design.

## Figures and Tables

**Table 1:** Measured grain sizes of 304 SS sheets under various heat treatment temperatures (μm).

	*t*/mm	0.05	0.08	0.10	0.12	0.15
*T*/°C						
850		8 ± 1	18 ± 2	20 ± 1	26 ± 2	30 ± 3
900		11 ± 2	19 ± 2	24 ± 2	30 ± 3	35 ± 4
950		28 ± 3	29 ± 3	30 ± 2	39 ± 3	47 ± 5
1000		49 ± 5	50 ± 4	51 ± 4	55 ± 7	56 ± 5

**Table 2:** Thinning rate and springback angle under different structural parameters.

No.	*α*/°	*R*/mm	*W*/mm	*h*/mm	*S*/mm	TR/%	SA/°
1	15	0.15	0.5	1.2	0.7	5.91	0.28
2	10	0.15	0.5	1.3	0.8	7.05	0.14
3	10	0.15	0.6	1.3	0.8	9.82	0.30
4	15	0.3	0.55	1.4	1.1	4.77	0.62
5	5	0.1	0.45	1.3	0.8	10.96	0.53
6	5	0.15	0.6	1.3	0.8	10.97	0.64
7	15	0.1	0.55	1.4	0.9	12.33	0.21
8	15	0.3	0.65	1.5	1	2.97	0.57
9	15	0.2	0.45	1.3	0.9	4.86	0.25
10	15	0.3	0.6	1.2	0.9	4.77	0.43
11	5	0.1	0.45	1.5	1.2	41.05	0.57
12	20	0.25	0.5	1.2	0.8	3.48	0.49
13	15	0.2	0.45	1.5	1.1	5.17	0.47
14	20	0.15	0.5	1.5	1	5.77	0.65
15	5	0.15	0.65	1.5	1.2	36.74	0.79
16	20	0.15	0.6	1.5	1	7.86	0.37
17	25	0.15	0.45	1.5	1.2	7.77	0.43
18	5	0.15	0.6	1.5	1.2	19.97	0.68
19	15	0.2	0.5	1.3	0.9	5.54	0.32
20	10	0.25	0.5	1.5	1.2	6.65	0.37
21	20	0.15	0.5	1.3	1	9.61	0.84
22	15	0.2	0.5	1.4	1	5.76	0.40
23	10	0.25	0.6	1.2	0.8	5.47	0.37
24	15	0.2	0.55	1.2	0.9	8.18	0.41
25	10	0.25	0.5	1.5	1.1	5.11	0.15
26	20	0.15	0.6	1.3	1	11.58	0.35
27	20	0.25	0.6	1.2	0.8	4.44	0.45
28	5	0.25	0.6	1.3	0.9	6.00	0.98
29	15	0.2	0.6	1.4	1	7.70	0.53
30	20	0.25	0.55	1.2	0.9	5.10	0.44
31	5	0.2	0.55	1.4	0.9	6.20	0.37
32	10	0.15	0.6	1.5	1.2	15.81	0.44
33	10	0.25	0.6	1.3	0.9	5.65	0.31
34	15	0.25	0.65	1.3	1	7.63	0.47
35	10	0.15	0.5	1.5	1.2	11.52	0.34
36	25	0.25	0.65	1.3	0.9	4.51	0.57
37	20	0.25	0.6	1.4	1.1	6.73	0.59
38	25	0.1	0.5	1.5	1.2	13.72	0.65
39	15	0.2	0.55	1.4	1.1	8.57	0.52
40	5	0.25	0.65	1.5	1.1	7.67	0.88
41	25	0.15	0.65	1.5	1.2	11.94	0.35

**Table 3:** Comparison of optimal process parameters and corresponding responses obtained using the RF-DE and XGBoost-DE models.

	*α*	*R*	*W*	*h*	*S*	Predicted Thinning Rate (*δ*)	Predicted Springback Angle (*f*)
Random Forest + Differential Evolution	16.03	0.30	1.46	0.48	0.73	4.00	0.14
XGBoost + Differential Evolution	17.21	0.26	1.26	0.45	0.80	5.50	0.14

**Table 4:** Comparison of mean absolute error (MAE) between the RF and XGBoost surrogate models.

	Train Set		Test Set	
	Thinning Rate (*δ*)	Springback Angle (*f*)	Thinning Rate (*δ*)	Springback Angle (*f*)
Random Forest + Differential Evolution	2.96	0.38	1.56	0.94
XGBoost + Differential Evolution	1.64	0.58	2.01	0.46

**Table 5:** Comparison of simulated results of FEM with different structural parameters.

*α*	*R*	*W*	*h*	*S*	Simulated Thinning Rate (*δ*)	Simulated Springback Angle (*f*)
16	0.30	1.46	0.48	0.73	4.43	0.151
17	0.26	1.26	0.45	0.80	6.31	0.155

## Data Availability

The raw data supporting the conclusions of this article will be made available by the authors on request.
